# Papillary thyroid microcarcinoma with multiple pulmonary metastases following lung cancer surgery: a case report

**DOI:** 10.1186/s40792-022-01570-y

**Published:** 2022-12-08

**Authors:** Hidenori Kamio, Hiromi Onizuka, Yusaku Yoshida, Yoko Omi, Tamami Isaka, Yoji Nagashima, Kiyomi Horiuchi, Takahiro Okamoto

**Affiliations:** 1 Department of Endocrine Surgery, Tokyo Women’s Medical University, 8-1 Kawada-Cho, Shinjuku-Ku, Tokyo, Japan; 2 Department of Surgical Pathology, Tokyo Women’s Medical University, 8-1 Kawada-Cho, Shinjuku-Ku, Tokyo, Japan; 3 Department of Thoracic Surgery, Tokyo Women’s Medical University, 8-1 Kawada-Cho, Shinjuku-Ku, Tokyo, Japan

**Keywords:** Low-risk papillary thyroid microcarcinoma (PTMC), Active surveillance (AS), Distant metastasis

## Abstract

**Background:**

Distant metastasis is extremely rare for papillary thyroid microcarcinoma (PTMC) without lymph node metastasis or extrathyroidal extension, for which active surveillance (AS) is indicated. The evaluation of distant metastases in low-risk PTMC is controversial. A case of PTMC in which AS would have been performed if chest CT and lung surgery had not been performed is reported.

**Case presentation:**

The patient was a 71-year-old woman undergoing follow-up in the Department of Thoracic Surgery at our hospital for multiple frosted glass shadows in both lung fields for one and a half years. To make a definitive diagnosis, thoracoscopic right middle lobectomy and left upper partial lobectomy were performed 4 and 6 months earlier, respectively. In both resected specimens, lung adenocarcinoma and small metastasis of papillary thyroid carcinoma (PTC) were found. The patient was transferred to our department for a thorough examination for PTC. Ultrasonography was performed to search for the primary lesion, and it showed an irregular hypoechoic mass of 4 mm and 6 mm in the middle of the right lobe of the thyroid gland. The patient was diagnosed with PTC. Its clinical stage was T1a (m) N0 M1 (stage IVC). Total thyroidectomy and prophylactic central node dissection were performed. The pathological diagnosis was PTC (typical type) pT1a (m) N0. Postoperatively, she received radioactive iodine therapy.

**Conclusions:**

We experienced an extremely rare case and struggled to determine a treatment plan. We might be aware that lung metastases could develop in low-risk PTMC.

## Background

The Japan Association of Endocrine Surgery (JAES) published clinical practice guidelines on the management of thyroid tumors in 2018. It recommends active surveillance (AS) for low-risk (cT1a N0 M0) papillary thyroid microcarcinoma (PTMC) if the patient wishes to have non-operative and follow-up care after receiving a full explanation [1]. The Japan Thyroid Association published a position paper on the management of low- risk papillary thyroid microcarcinoma in adults, which defines low-risk PTMC as PTMC without clinically apparent metastasis and invasion [2]. Distant metastasis is assumed to be rare in low-risk PTMC; no reports found new distant metastasis during AS [2–4]. Therefore, it is controversial whether to search for distant metastasis in PTMC eligible for AS [1, 2].

Here, we report a case of low-risk PTMC in which AS would have been performed if the lung surgery had not revealed micro lung metastasis coincidentally.

## Case presentation

**Fig. 1:**
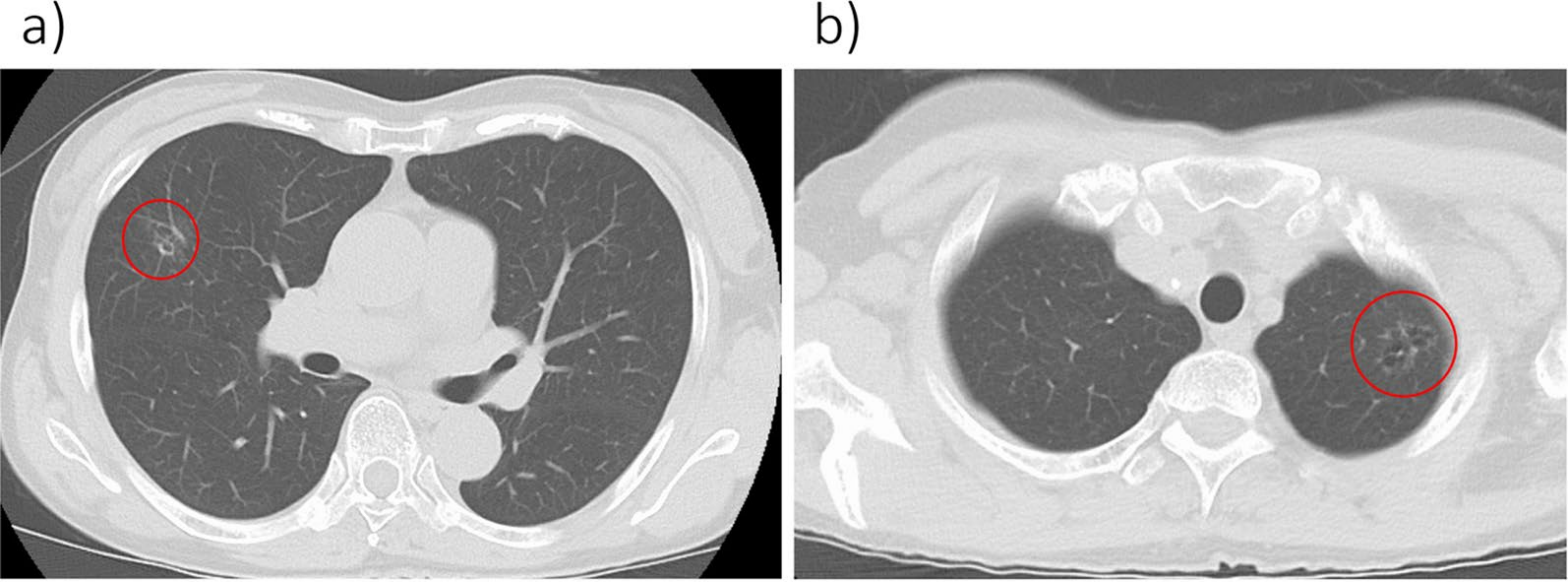
Computed tomography findings of the lung tumor. **a** A frosted glass shadow is observed in the middle lobe of the right lung. **b** A frosted glass shadow is observed in the upper lobe of the left lung

The patient was a 71-year-old woman undergoing follow-up in the Department of Thoracic Surgery at our hospital for multiple frosted glass shadows suggestive of primary lung carcinoma in both lung fields for one and a half years (Fig. 1a, b). On computed tomography, there were no lung nodules suggestive of metastatic tumor. Although there were no apparent changes over time, it was decided to perform surgery for a definitive diagnosis because the patient wished it. Thoracoscopic right middle lobectomy and left upper partial lobectomy were performed 4 and 6 months earlier, respectively.

**Fig. 2:**
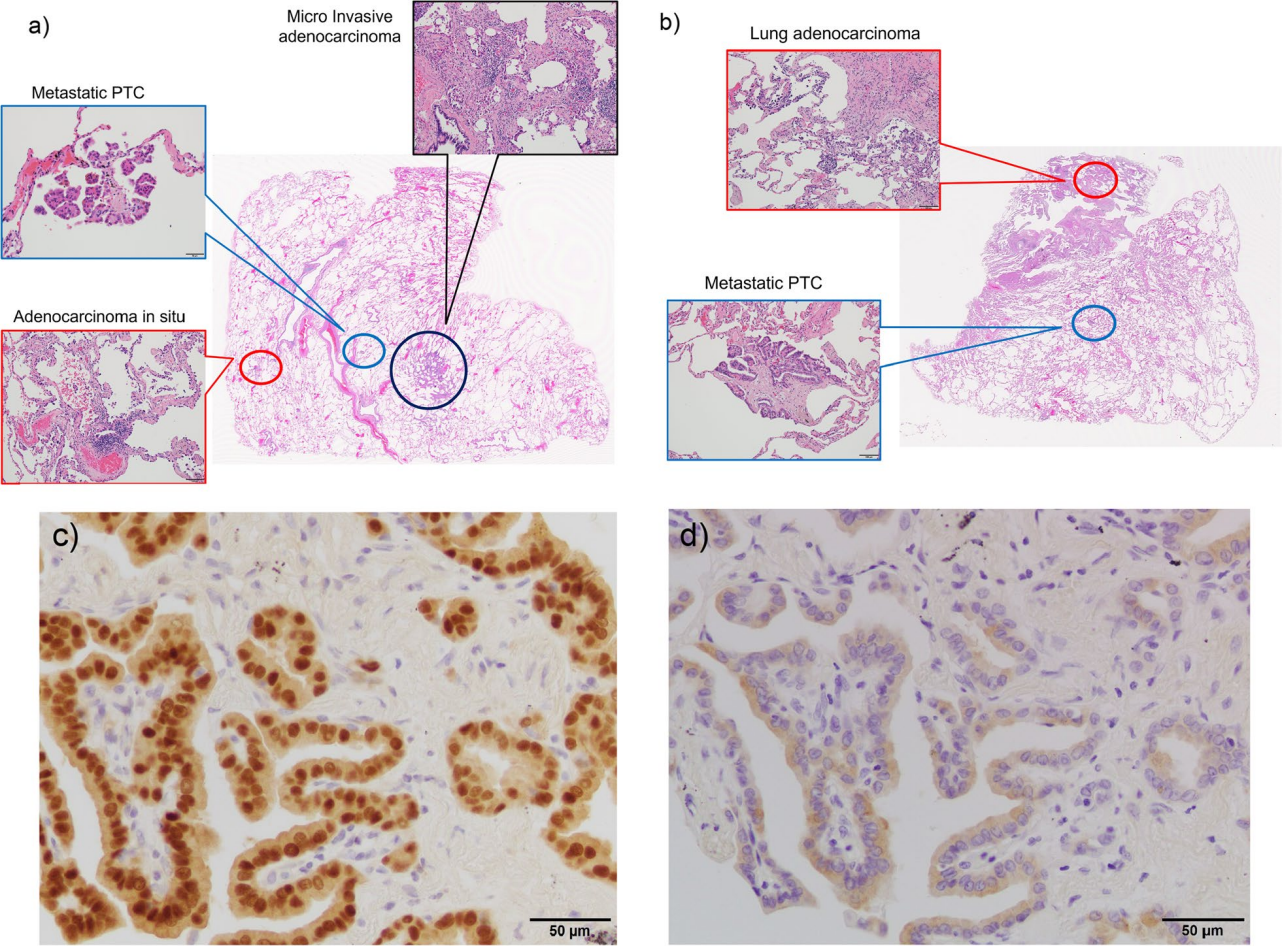
Pathological findings of the lung tumor. **a** A loupe image of the right lung specimen (H&E staining). This shows the location of lung adenocarcinoma (× 100), adenocarcinoma in situ (× 100) and metastatic PTC (× 200). **b** A loupe image of the left lung specimen (H&E staining). This shows the location of lung adenocarcinoma (× 100) and metastatic PTC (× 100). **c**, **d** The cells of metastatic PTC are positive for **c** PAX8 and **d** thyroglobulin.

The size of the resected specimen of the right lung was 13 × 8.5 × 4 cm. In the specimen, there were minimally invasive lung adenocarcinoma with an invasive diameter of 7 mm, adenocarcinoma in situ in two locations, and microscopic metastatic PTC in four locations. The size of the resected specimen of the left lung was 8 × 3 × 3.5 cm. In the specimen, there were a lung adenocarcinoma with an invasive diameter of 9 mm and a metastatic PTC 0.5 mm in size at the same site. In both resected specimens, the cells of metastatic PTC were positive for PAX8 and thyroglobulin. These findings were consistent with PTC (Fig. 2). The patient came to our department for a thorough examination for PTC.

The serum thyroid-stimulation hormone (TSH), free triiodothyronine (fT3), free thyroxine (fT4), and thyroglobulin levels were 2.60 mIU/L, 2.15 pg/ml, 1.60 ng/dl, and 22.8 ng/ml, respectively. Anti-thyroglobulin antibodies were negative.

**Fig. 3:**
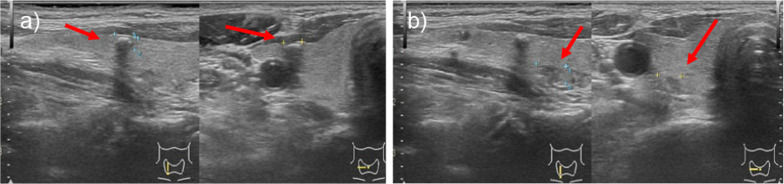
Ultrasonographic findings of the thyroid tumors. **a** A hypoechoic mass with rim calcification, 4 mm in size, is found in the middle of the right lobe of the thyroid gland. **b** An irregular hypoechoic mass, 6 mm in size, is found in the middle of the right lobe of the thyroid gland

**Fig. 4:**
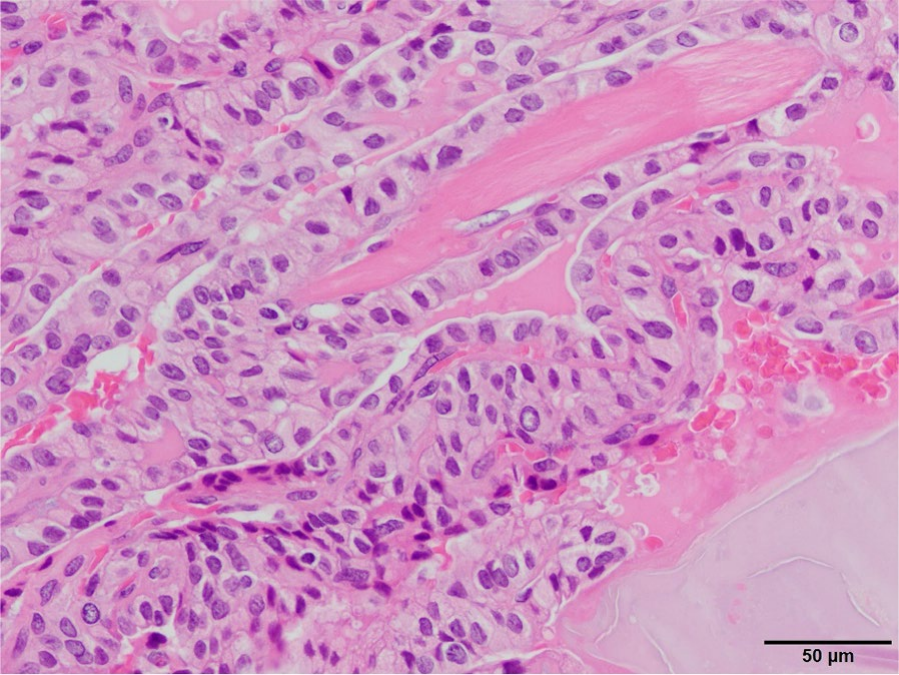
Pathological findings of the thyroid tumor. Papillary carcinoma with microinvasion in the central portion of the right thyroid lobe. There is no infiltration of the surrounding connective tissue

Ultrasonography was performed to search for the primary lesion and demonstrated an irregular hypoechoic mass of 4 mm and 6 mm in the middle of the right lobe of the thyroid gland. The fine-needle aspiration cytology from the 4 mm lesion revealed PTC (Fig. 3a). Preoperatively, the patient was diagnosed as cT1a (m) N0 M1 (stage IVB). Total thyroidectomy and prophylactic central node dissection were performed. The pathological diagnosis was PTC (typical type) pT1a (m) N0 (Fig. 4).

Postoperative radioiodine therapy was performed five months after the surgery. The patient was administered 100 mCi of 131 iodine (I-131), and the accumulation of I-131 in the thyroid bed was confirmed, though there was no accumulation in the lung field. Subsequently, at seven-month follow-up, the I-131 whole-body scan was negative. The thyroglobulin level during TSH stimulation and three months after RAI were 7.71 and 1.0 ng/ml.

## Discussion

A case of low-risk PTMC eligible for AS but caused multiple lung metastases without being identified on imaging was reported.

Although distant metastasis can occur even in microcarcinoma, it is thought to be associated with high-risk PTMC with clinically evident lymph node metastasis or extrathyroidal extension [1, 2]. Since there have been no reports of lung metastases at the diagnosis of low-risk PTMC, the JAES recommends that chest CT is not mandatory to search for distant metastases at the diagnosis of low-risk PTMC and during AS [5–8]. There are also no reports of the appearance of new distant metastasis in low-risk PTMC during AS [3, 4, 9]. On the other hand, there are many reports of occult PTC. Most of them were first detected with neck lymph node metastasis [10]. The occult PTC cases diagnosed after lung metastasis are extremely rare [11–13]. The previous papers have also reported cases of occult carcinoma with distant metastasis, including the case of pulmonary metastasis [14]. However, the lung metastases were more than 1 cm in diameter in all the previous reports and were easy to recognize. On the other hand, our case had no gross-visualized lung metastases, just frosted glass shadows which could not be diagnosed as malignant. To the best of our knowledge, distant metastasis after surgery for low-risk PTMC is extremely rare, with only one case of metastasis to skeletal muscle [15]. The present case may be the first reported case of multiple “occult” or “micro” lung metastases from low-risk PTMC that was eligible for AS.

If PTC had been detected first, it would have been difficult to diagnose the lung metastases on imaging, and this case would have likely been subjected to AS as cT1a N0 M0. By chance, metastases of PTC were found in the lung cancer lesion, which led to the diagnosis of high-risk PTC and allowed us to administer appropriate treatment, total thyroidectomy followed by radioactive iodine (RAI) treatment, in contrast to AS. Although the clinical practice guideline of the JAES and the Japan Thyroid Association task force defines cT1aN0M0 cases as low-risk PTMC, they do not mention a method to confirm M0 [1, 2].

The clinical practice guidelines of the JAES recommend postoperative RAI therapy for high-risk PTC, especially for lung metastases [16]. RAI is effective when it accumulates in microscopic foci that are not visible on imaging [17–21]. In the present case, diffuse accumulation of I-131 in the lung field was expected before therapy, considering the detection of lung metastases. Unexpectedly, no accumulation was observed.

As a treatment strategy for this case, several options were considered before thyroid surgery. One option was to limit the procedure to a right lobectomy of the thyroid gland, considering that the lung metastases might not affect the prognosis, since the lesion was too small to be visualized, and the patient was elderly. As another option, it was thought that AS could have been performed, since it was a small cancer, and the lung metastasis was too small to visualize. In reality, however, the patient chose total thyroidectomy plus RAI treatment. Considering the patient’s anxiety due to the discovery of lung metastases, we believe that this choice was appropriate.

## Conclusions

We experienced an extremely rare case and struggled to determine a treatment plan. We might be aware that lung metastases could develop in low-risk PTMC.

## Data Availability

All data generated or analyzed during this study are included in this published article.

## Abbreviations

JAES: Japan Association of Endocrine Surgery
AS: Active surveillance
PTMC: Papillary thyroid microcarcinoma
PTC: Papillary thyroid carcinoma
I-131: 131 Iodine
RAI: Radioactive iodine

## References

[1] Sugitani I, Ito Y, Takeuchi D, Nakayama H, Masaki C, Shindo H (2021). Indications and strategy for active surveillance of adult low-risk papillary thyroid microcarcinoma: consensus statements from the Japan Association of Endocrine Surgery Task Force on management for papillary thyroid microcarcinoma. Thyroid.

[2] Horiguchi K, Yoshida Y, Iwaku K, Emoto N, Kasahara T, Sato J (2021). Position paper from the Japan Thyroid Association Task Force on the management of low-risk papillary thyroid microcarcinoma (T1aN0M0) in adults. Endocr J.

[3] Sugitani I, Toda K, Yamada K, Yamamoto N, Ikenaga M, Fujimoto Y (2010). Three distinctly different kinds of papillary thyroid microcarcinoma should be recognized: Our treatment strategies and outcomes. World J Surg.

[4] Ito Y, Miyauchi A, Kihara M, Higashiyama T, Kobayashi K, Miya A (2014). Patient age is significantly related to the progression of papillary microcarcinoma of the thyroid under observation. Thyroid.

[5] Sugitani I, Fujimoto Y (1999). Symptomatic versus asymptomatic papillary thyroid microcarcinoma: a retrospective analysis of surgical outcome and prognostic factors. Endocr J.

[6] Ito Y, Uruno T, Nakano K, Takamura Y, Miya A, Kobayashi K (2003). An observation trial without surgical treatment in patients with papillary microcarcinoma of the thyroid. Thyroid.

[7] Choi JB, Lee WK, Lee SG, Ryu H, Lee CR, Kang SW (2018). Long-term oncologic outcomes of papillary thyroid microcarcinoma according to the presence of clinically apparent lymph node metastasis: a large retrospective analysis of 5,348 patients. Cancer Manag Res.

[8] Reinke R, Mathiesen JS, Larsen SR, Hahn CH, Pedersen HB, Bentzen J (2019). Incidental and non-incidental papillary thyroid microcarcinoma in Denmark 1996–2015: a national study on incidence, outcome and thoughts on active surveillance. Cancer Epidemiol.

[9] Tuttle RM, Fagin JA, Minkowitz G, Wong RJ, Roman B, Patel S (2017). Natural history and tumor volume kinetics of papillary thyroid cancers during active surveillance. JAMA Otolaryngol Head Neck Surg.

[10] Ito Y, Hirooka M, Fukushima M, Inoue H, Yabuta T, Uruno T (2008). Occult papillary thyroid carcinoma: diagnostic and clinical implications in the era of routine ultrasonography. World J Surg.

[11] Cavazza A, Roggeri A, Zini M, Rossi G, Zucchi L (2002). Lymphangioleiomyomatosis associated with pulmonary metastasis from an occult papillary carcinoma of the thyroid: report of a case occurring in a patient without tuberous sclerosis. Pathol Res Pract.

[12] Kaseda K, Watanabe K, Sakamaki H, Kazama A (2016). Solitary pulmonary metastasis from occult papillary thyroid carcinoma. Thorac Cancer.

[13] Itoh M, Oh-ishi S, Senba S, Nemoto K, Hatao H, Shimizudani N (2008). A case of occult thyroid cancer detected as a solitary nodular lung metastasis. Nihon Kokyuki Gakkai Zasshi.

[14] Shimizu T, Oba T, Chino T, Soma A, Ono M, Ito T (2021). Papillary thyroid microcarcinoma with lung metastases: a case report and review of the literature. Thyroid Res.

[15] Sarma M, Sonik B, Subramanyam P, Sundaram PS (2015). Isolated skeletal muscle metastatic deposit in a patient with micropapillary carcinoma thyroid identified by 18F FDG PET CT. J Egypt Natl Canc Inst.

[16] Ito Y, Onoda N, Okamoto T (2020). The revised clinical practice guidelines on the management of thyroid tumors by the Japan Associations of Endocrine Surgeons: core questions and recommendations for treatments of thyroid cancer. Endocr J.

[17] Schlumberger M, Challeton C, De Vathaire F, Travagli JP, Gardet P, Lumbroso JD (1996). Radioactive iodine treatment and external radiotherapy for lung and bone metastases from thyroid carcinoma. J Nucl Med.

[18] Ilgan S, Karacalioglu AO, Pabuscu Y, Atac GK, Arslan N, Ozturk E (2004). Iodine-131 treatment and high-resolution CT: results in patients with lung metastases from differentiated thyroid carcinoma. Eur J Nucl Med Moi Imaging.

[19] Hod N, Hagag P, Baumer M, Sandbank J, Horne T (2005). Differentiated thyroid carcinoma in children and young adults: evaluation of response to treatment. Clin Nucl Med.

[20] Durante C, Haddy N, Baudin E, Leboulleux S, Hartl D, Travagli JP (2006). Long-term outcome of 444 patients with distant metastases from papillary and follicular thyroid carcinoma: benefits and limits of radioiodine therapy. J Clin Endocrinol Metab.

[21] Song HJ, Qiu ZL, Shen CT, Wei WJ, Luo QY (2015). Pulmonary metastases in differentiated thyroid cancer: efficacy of radioiodine therapy and prognostic factors. Eur J Endocrinol.

